# Clinical impact of left and right axis deviations with narrow QRS complex on 3-year outcomes in a hospital-based population in Japan

**DOI:** 10.1038/s41598-021-88259-8

**Published:** 2021-04-26

**Authors:** Yuta Seko, Takao Kato, Yuhei Yamaji, Yoshisumi Haruna, Eisaku Nakane, Tetsuya Haruna, Moriaki Inoko

**Affiliations:** 1 Department of Cardiovascular Medicine, Kyoto University Graduate School of Medicine, 54 Shogoin Kawahara-cho, Sakyo-ku, Kyoto, 606-8507 Japan; 2 Cardiovascular Center, Kitano Hospital, The Tazuke Kofukai Medical Research Institute, Osaka, Japan

**Keywords:** Cardiology, Medical research, Outcomes research

## Abstract

While the prognostic impact of QRS axis deviation has been assessed, it has never been investigated in patients without conduction block. Thus, we evaluated the prognostic impact of QRS-axis deviation in patients without conduction block. We retrospectively analyzed 3353 patients who had undergone both scheduled transthoracic echocardiography and electrocardiography in 2013 in a hospital-based population, after excluding patients with a QRS duration of ≥ 110 ms, pacemaker placement, and an QRS-axis − 90° to − 180° (northwest axis). The study population was categorized into three groups depending on the mean frontal plane QRS axis as follows: patients with left axis deviation (N = 171), those with right axis deviation (N = 94), and those with normal axis (N = 3088). The primary outcome was a composite of all-cause death and major adverse cardiovascular events. The cumulative 3-year incidence of the primary outcome measure was significantly higher in the left axis deviation group (26.4% in the left axis deviation, 22.7% in the right axis deviation, and 18.4% in the normal axis groups, log-rank P = 0.004). After adjusting for confounders, the excess risk of primary outcome measure remained significant in the left axis deviation group (hazard ratio [HR] 1.44; 95% confidence interval [CI] 1.07–1.95; P = 0.02), while the excess risk of primary outcome measure was not significant in the right axis deviation group (HR 1.22; 95% CI 0.76–1.96; P = 0.41). Left axis deviation was associated with a higher risk of a composite of all-cause death and major adverse cardiovascular events in hospital-based patients without conduction block in Japan.

## Introduction

QRS axis deviation can be easily determined in electrocardiograms (ECGs). Although left axis deviation is often an age-related physiological change^1–3^, it may indicate the presence of various conditions, such as left ventricular hypertrophy^4^, left anterior fascicular block^5^, inferior wall myocardial infarction^6^, emphysema^7^, and mechanical shift due to elevated diaphragm because of obesity^8^. Right axis deviation is usually seen in children and young adults under physiological conditions^3,9^. However, most causes of pathological conditions in right axis deviation are right ventricular hypertrophy^10^, lateral myocardial infarction^11^, altered conduction pathways^5^, and changes in the position of the heart in the chest^12^.

While the prognostic impact of QRS axis deviation in patients including a wide QRS complex has been assessed from the 1980s in Western countries^11,13–15^, it has never been investigated in patients without conduction block in Asian population. Thus, the present study investigated the prognostic impact of QRS axis deviation with QRS duration less than 110 ms and performed multivariable adjustment to assess the adjusted risk of QRS axis deviation.

## Methods

### Study design, setting, and population

**Figure 1:**
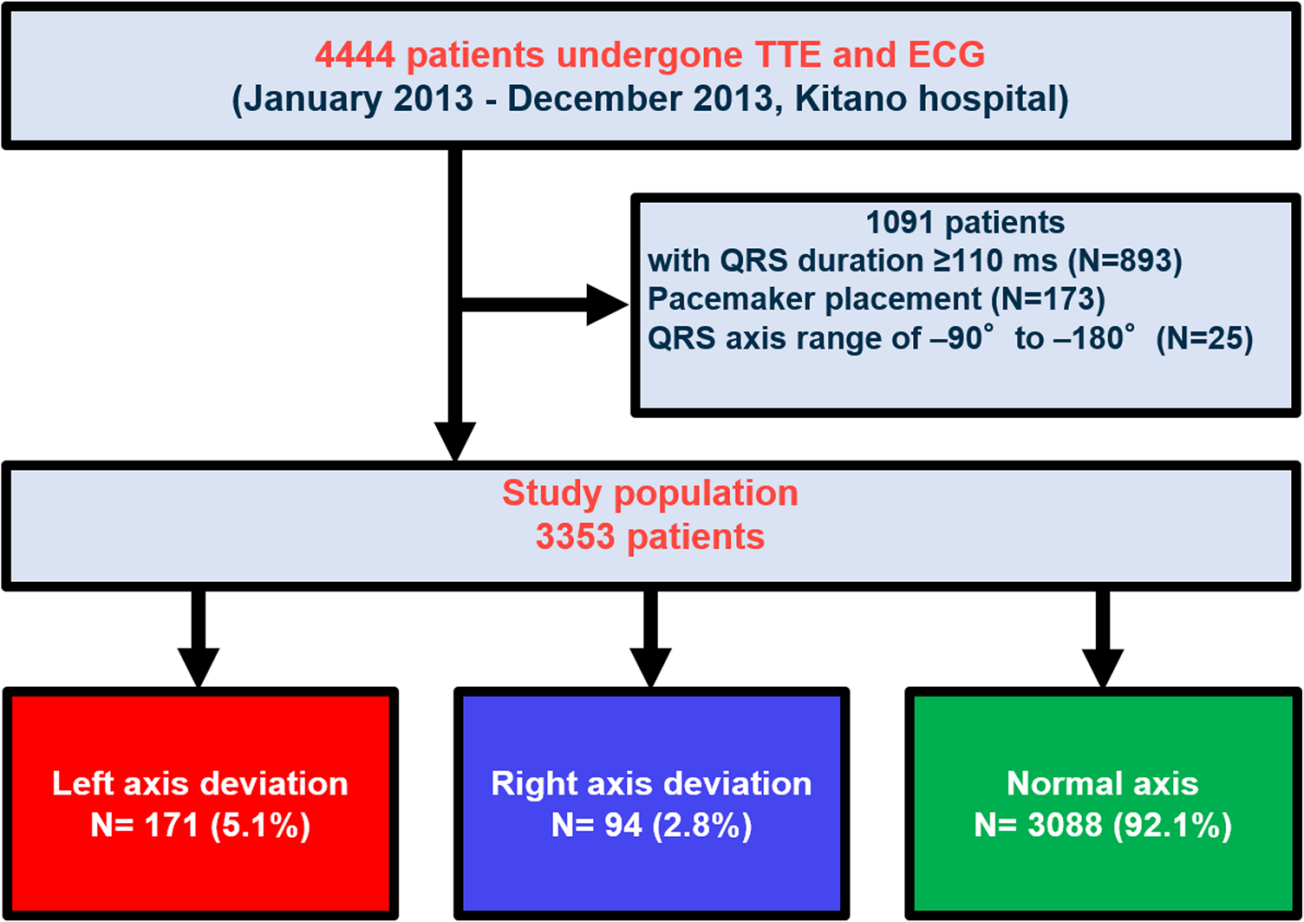
Patient flowchart. *TTE* transthoracic echocardiography, *ECG* electrocardiogram.

We retrospectively analyzed 4444 patients who had undergone simultaneous scheduled transthoracic echocardiography and electrocardiography at outpatient visits or stable in-hospital conditions at Kitano Hospital in 2013, which were ordered at the physician’s discretion. A flowchart of the study population is shown in Fig. 1. We excluded 1091 patients who did not meet the criteria for follow-up, QRS duration ≥ 110 ms (N = 893), pacemaker placement (N = 173), and QRS-axis range of − 90° to − 180° (northwest axis) (N = 25). The study population comprised 3353 patients, who were categorized into three groups depending on the mean frontal plane QRS-axis as follows: left axis deviation (− 30° to − 90°), right axis deviation (90° to 180°), and normal axis (− 30° to + 90°). We used the AHA/ACCF/HRS recommendation of ≥ 110 ms QRS duration as a wide QRS complex definition^16^.

As a supplementary analysis, we also adopted Japanese recommendation of ≥ 100 ms QRS duration as a wide QRS complex definition^17^. A flowchart of the study population using QRS ≥ 100 ms as exclusion criteria is shown in Supplementary Fig. 1.

### Ethics

The research protocol was approved by the Institutional Review Board of Kitano Hospital (approval no. P16-02-005)^18,19^. The requirement of informed consent was waived because of the retrospective study design. We disclosed the details of the present study to the public as an opt-out method and the notice clearly informed patients of their right to refuse enrollment. The study protocol conformed to the ethical guidelines of the 1975 Declaration of Helsinki, as reflected in a priori approval by the institution’s Human Research Committee. Patient records and information were anonymized and deidentified before analysis.

### Data collection

Using the ECG database, we extracted data regarding QRS duration and the mean frontal plane QRS axis^18^. Using the transthoracic echocardiography database, we extracted data regarding wall thickness, left ventricular (LV) diastolic dimensions, LV systolic dimensions, left atrial dimension, left atrial volume index, LV ejection fraction, transmitral flow, tissue Doppler imaging, and body mass index (BMI)^19–24^. Based on the transthoracic echocardiography data along with the catheter suite database, we identified patients with previous myocardial infarction or structural heart disease. LV mass index and relative wall thickness were calculated using the formula recommended by the American Society of Echocardiography^25^. A high LV mass index was defined as LV mass index > 115 g/m^2^ for men and > 95 g/m^2^ for women. The LV ejection fraction was measured using the Teichholz or modified Simpson’s rule methods. All transthoracic echocardiography measurements were performed using the average of at least three cardiac cycles. We also extracted patient information from their electronic medical records at our institution, including age, sex, and type of disease (i.e., ischemic heart disease, International Statistical Classification of Diseases and Related Health Problems, Tenth Revision [ICD-10] codes I20, I21, I22, I23, I24, and I25; hypertension, ICD-10 codes I10, I11, I12, I13, I14, and I15; dyslipidemia, ICD-10 code E78; diabetes mellitus, ICD-10 codes E10, E11, E12, E13, and E14; and chronic kidney disease, ICD-10 code N18)^18,19^. Follow-up data from serial clinic visits until June 2017 were also collected retrospectively from electronic medical records.

### Outcome measures

The primary outcome measure was a composite of all-cause death and major adverse cardiovascular events (MACE) defined as acute heart failure, acute myocardial infarction, unstable angina pectoris, cerebral infarction, cerebral hemorrhage, and emerging aorta and peripheral vascular disease, including treatment for aortic aneurysm, all of which required unplanned hospitalization. The secondary outcome measures were all-cause death and MACE, separately.

### Statistical analysis

Categorical variables are presented as numbers with percentages and were compared using the chi square test or Fisher’s exact tests. Continuous variables are expressed as means with standard deviations and were compared using one-way analysis of variance (ANOVA) tests. We compared the patient characteristics and 3-year clinical outcomes among the three groups with (1) left axis deviation, (2) right axis deviation, and (3) normal axis. The cumulative incidences of clinical events were estimated using the Kaplan Meier method, and intergroup differences were assessed using the log-rank tests. Multivariable Cox proportional hazards models were used to estimate the risk of primary and secondary outcomes associated with the three groups. The model also included the following nine clinically relevant covariates: age > 70 years, BMI > 25 kg/m^2^, diabetes, hypertension, dyslipidemia, ischemic heart disease, chronic kidney disease, LV ejection fraction < 50%, and high LV mass index. In a supplementary analysis, we used the Japanese criteria which define wide QRS complex as a QRS duration of ≥ 100 ms^17^ and excluded patients with QRS ≥ 100 ms from the analysis. The results are expressed as hazard ratios (HRs) and 95% confidence intervals (CIs). All statistical analyses were performed by physicians (YS and TK) using JMP 15. All reported P values are two-tailed and the level of statistical significance was set at P < 0.05.

## Results

### Baseline clinical and echocardiographic characteristics

This study included 171 patients (5.1%) with left axis deviation, 94 patients (2.8%) with right axis deviation, and 3088 patients (92.1%) with normal axis (Fig. 1). The baseline characteristics of the study population are presented in Table 1. The patients with left axis deviation were older, were more often men, and were more likely to have hypertension, dyslipidemia, ischemic heart disease, and a high LV mass index than those with normal axis (Table 1). The patients with right axis deviation were younger and more likely to have atrial fibrillation. The baseline characteristics using QRS ≥ 100 ms as exclusion criteria are presented in Supplementary Table 1.

**Table 1:** Baseline characteristics of the study subjects and transthoracic echocardiography results of the patients.

	Total (n = 3353)	Left axis deviation (n = 171)	Right axis deviation (n = 94)	Normal axis (n = 3088)	P value	Total
Age, years	65.6 ± 15.7	72.7 ± 11.5	60.6 ± 17.2	65.4 ± 15.8	< 0.001	3353
Age > 70 years*	1534 (45.8)	107 (62.6)	33 (35.1)	1394 (45.1)	< 0.001	3353
Women	1695 (50.6)	71 (41.5)	51 (54.3)	1573 (50.9)	0.04	3353
Body mass index (BMI) kg/m^2^	23.1 ± 4.3	22.3 ± 3.7	21.6 ± 4.5	23.2 ± 4.3	< 0.001	3333
BMI > 25 kg/m^2^*	918 (27.5)	35 (20.7)	16 (17.2)	867 (28.2)	0.008	3333
Atrial fibrillation	336 (10.0)	14 (8.2)	19 (20.2)	303 (9.8)	0.003	3353
Diabetes*	992 (29.6)	55 (32.2)	23 (24.5)	914 (29.6)	0.42	3353
Hypertension*	1905 (56.8)	112 (65.5)	41 (43.6)	1752 (56.7)	0.003	3353
Dyslipidemia*	944 (28.2)	63 (36.8)	21 (22.3)	860 (27.9)	0.02	3353
Ischemic heart disease*	1016 (30.3)	85 (49.7)	29 (30.9)	902 (29.2)	< 0.001	3353
Chronic kidney disease*	468 (14.0)	30 (17.5)	7 (7.5)	431 (14.0)	0.08	3353
LVDd, cm	4.63 ± 0.56	4.67 ± 0.60	4.54 ± 0.63	4.63 ± 0.56	0.19	3353
LVDs, cm	3.09 ± 0.51	3.13 ± 0.56	3.06 ± 0.56	3.09 ± 0.50	0.47	3353
IVSTd, cm	0.81 ± 0.16	0.85 ± 0.17	0.79 ± 0.16	0.81 ± 0.16	0.003	3353
LVPWd, cm	0.80 ± 0.14	0.83 ± 0.14	0.77 ± 0.16	0.80 ± 0.14	< 0.001	3353
RWT	0.35 ± 0.07	0.36 ± 0.07	0.34 ± 0.07	0.35 ± 0.07	0.03	3353
LVMI, g/m^2^	76.6 ± 22.7	83.9 ± 24.5	73.2 ± 27.4	76.3 ± 22.3	< 0.001	3342
High LVMI*	354 (10.6)	29 (17.0)	9 (9.7)	316 (10.3)	0.02	3342
LAVI, ml/m^2^	23.4 ± 13.3	25.4 ± 16.7	26.9 ± 25.9	23.2 ± 12.5	0.009	3019
EF, %	61.9 ± 7.1	60.6 ± 8.0	60.9 ± 8.2	62.0 ± 7.0	0.02	3353
EF < 50%*	198 (5.9)	20 (11.7)	9 (9.6)	169 (5.5)	0.001	3353
HR, bpm	72.2 ± 15.6	74.4 ± 17.1	76.6 ± 16.2	71.9 ± 15.5	0.003	3353

Values are number (%), mean ± SD.

*BMI* body mass index, *EF* ejection fraction, *HR* heart rate, *IVSTd* diastolic interventricular septal wall thickness, *LAVI* left atrial volume index, *LVDd* left ventricular diastolic dimension, *LVDs* left ventricular systolic dimension, *LVMI* left ventricular mass index, *LVPWd* diastolic left ventricular posterior wall thickness, *RWT* relative wall thickness.

P values were calculated using the chi square test or Fisher’s exact test for categorical variables, and the one-way analysis of variance (ANOVA) test for continuous variables.

*Potential risk-adjusting variables selected for cox proportional hazard model.

### Clinical outcomes of frontal plane QRS axis deviation

**Figure 2:**
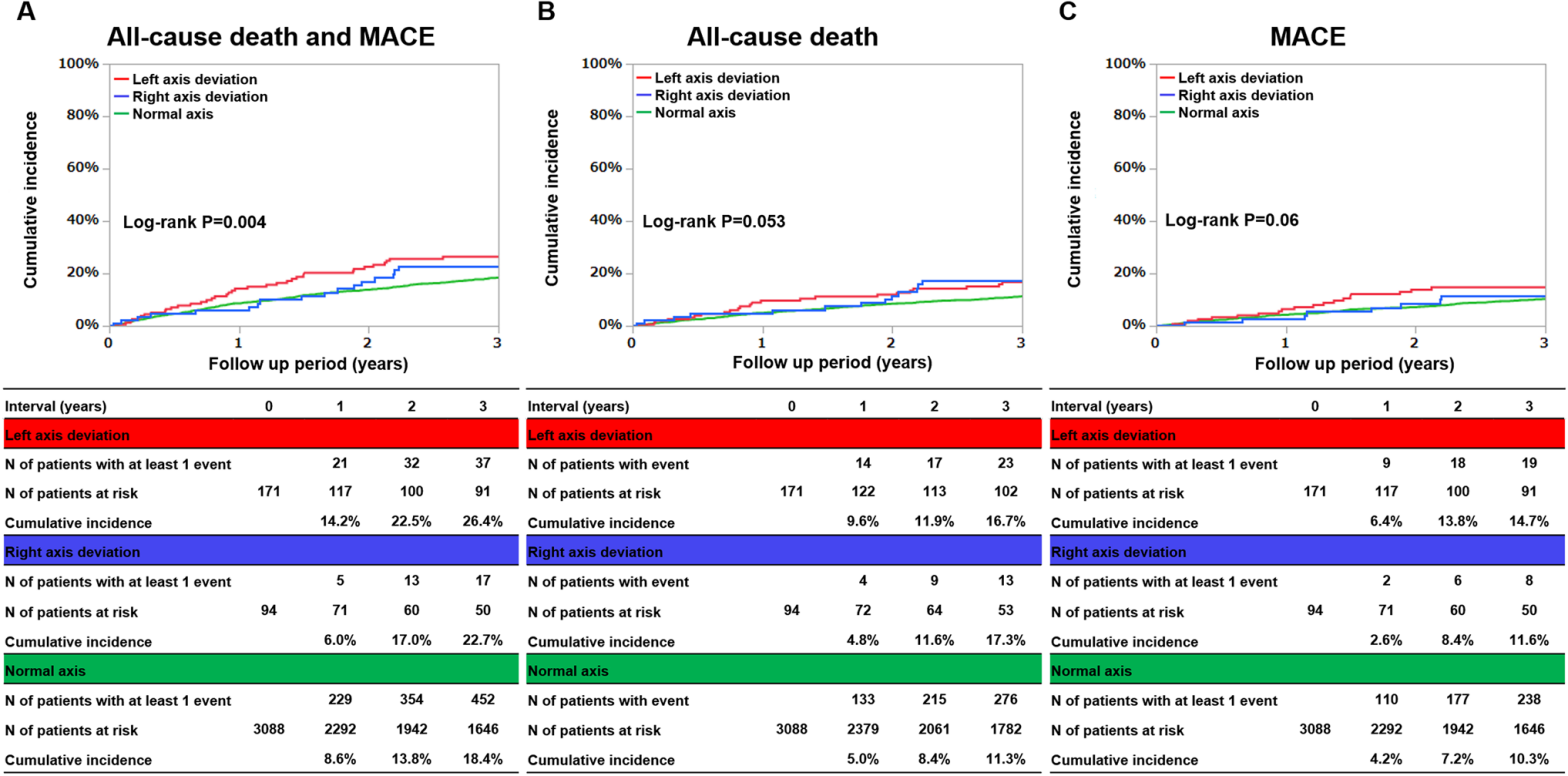
Cumulative incidence of the primary outcome measure (a composite of all-cause death and MACE) and secondary outcomes measure (all-cause death, MACE). (**A**) A composite of all-cause death and MACE, (**B**) all-cause death, (**C**) MACE. *MACE* major adverse cardiovascular events.

The median follow-up duration was 1273 (IQR 426–1467) days, with a 80.4% follow-up rate at 1 year. The cumulative 3-year incidence of the primary outcome measure was significantly higher in the left axis deviation group (left axis deviation: 26.4%, right axis deviation: 22.7%, and normal axis: 18.4%: log-rank P = 0.004) (Fig. 2A). After adjusting for confounders, the excess risk of primary outcome measure remained significant in the left axis deviation group (HR 1.44; 95% CI 1.07–1.95; P = 0.02), while the excess risk of primary outcome measure was not significant in the right axis deviation group (Table 2). The cumulative 3-year incidences of secondary outcome measures did not differ significantly among the three groups (Fig. 2B,C). After adjusting for confounders, the excess risks of the secondary outcome measures, all-cause death and MACE, respectively, were not significant in the left axis deviation group (Table 2). The excess risks of the secondary outcome measures were not significant in the right axis deviation group (Table 2).

**Table 2:** Clinical outcomes of patients in each frontal plane QRS axis groups.

	Left axis deviation N of patients with event/N of patients at risk (Cumulative 3-year incidence [%])	Right axis deviation N of patients with event/N of patients at risk (Cumulative 3-year incidence [%])	Normal axis N of patients with event/N of patients at risk (Cumulative 3-year incidence [%])	Variables	Unadjusted		Adjusted	
					HR (95% CI)	P value	HR (95% CI)	P value
**Primary outcome**								
A composite of all-cause death and MACE	37/171 (26.4)	17/94 (22.7)	452/3088 (18.4)	Left axis deviation	1.64 (1.22–2.20)	0.001	1.44 (1.07–1.95)	0.02
				Right axis deviation	1.10 (0.69–1.76)	0.70	1.22 (0.76–1.96)	0.41
				Normal axis	Reference		Reference	
**Secondary outcomes**								
All-cause death	23/171 (16.7)	13/94 (17.3)	276/3088 (11.3)	Left axis deviation	1.52 (1.04–2.22)	0.03	1.44 (0.98–2.12)	0.06
				Right axis deviation	1.37 (0.80–2.34)	0.25	1.61 (0.94–2.76)	0.08
				Normal axis	Reference		Reference	
MACE	19/171 (14.7)	8/94 (11.6)	238/3088 (10.3)	Left axis deviation	1.62 (1.08–2.44)	0.02	1.28 (0.85–1.94)	0.24
				Right axis deviation	0.93 (0.46–1.87)	0.83	1.05 (0.52–2.14)	0.88
				Normal axis	Reference		Reference	

*CI* confidence interval, *HR* hazard ratio, *MACE* major adverse cardiovascular events.

### Supplementary analysis

When we used the Japanese criteria which define wide QRS complex as a QRS duration of ≥ 100 ms, the results were fully consistent with the main analysis (Supplementary Fig. 2, Supplementary Table 2).

## Discussions

The results of this study illustrated that (1) patients with left axis deviation with narrow QRS complex had worse clinical outcomes in hospital-based population in Japan after adjusting for confounders; (2) the outcomes in patients with right axis deviation and narrow QRS complex were not different compared to those in patients with normal QRS axis; and (3) the results were essentially same both using the AHA/ACCF/HRS recommendation of ≥ 110 ms QRS duration and the Japanese recommendation of ≥ 100 ms QRS duration of wide QRS complex definition. These are the novel findings of the present study.

O’Neal et al. reported that left and right axis deviations were independent risk factors related to heart failure with reduced LV ejection fraction in patients without cardiovascular disease^14^. This does not appear to be consistent with our results; however, the target populations differed between the two studies. O’Neal et al. reported that, while the patients had no apparent cardiovascular disease in the US, a substantial proportion of them showed the wide QRS complex (20%) and were having anti-hypertensive drugs (36%), statins (15%), and aspirin (24%), but without adjustment for the comorbid diseases. Our study comprised outpatient with cardiovascular risk factors or diseases in Japan. Moreover, we excluded the patients with wide QRS complex and performed the adjustment for comorbid diseases, LV ejection fraction, and LV mass index. Our data suggested that left axis deviation was an independent factor associated with worse outcomes in a hospital-based population with narrow QRS complex in Japan. Right axis deviation itself showed no apparent increased risk for outcomes in the present study. While there may be a serious condition that causes right axis deviation^5,10,11^, its risk in a hospital-based outpatient was comparable to that of the normal axis in the present study. This may be because a substantial proportion of the right axis deviation may be a normal variant considering the younger patients included.

Left axis deviation may be caused by various physiological and pathological conditions^1–7^. Hypertension along with LV hypertrophy causes elevated pressure in the LV cavity and affects the conduction system; it also results in a more horizontal heart position^4^. Due to the accumulation of abdominal organ fat, the obesity may be related to the more horizontal heart position through diaphragm elevation^8^. However, high BMI was more common in patients with normal QRS axis than in patients with left axis deviation in our study. This may be due to the result that patients with normal axis were significantly younger than those with left axis deviation. Older age may cause stiffening of the aorta, leading to the LV hypertrophy and changes in heart position^2^. All these factors have prognostic influences on the outcome. However, after adjusting for these factors, left axis deviation remained at risk for adverse outcomes. These results suggested two possible mechanisms why the left axis deviation was linked to worse clinical outcomes. First, left axis deviation reflect the horizontal heart position due to other unadjusted causes such as congenital heart disease or pulmonary disease^7^. Second, mild but unacknowledged multiple pathological conditions caused left axis deviation. In either case, we should try to look for comorbidities and unacknowledged conditions that can cause left axis deviation, if they exist, by systematic evaluation to prevent their worse clinical outcome. Because ECG is a simple and easy modality, the left axis deviation should signal the need for careful observation of given patients.

### Limitations

This study has several limitations. First, our data shows a significant difference in the primary outcome but fails to show a difference in the secondary outcomes. Although the trend of the secondary outcomes was consistent across the groups, the reason why there was no significant difference in the secondary outcomes may be due to the low incidence rate of events in stable patients who underwent scheduled tests. Second, ECG and transthoracic echocardiograms were ordered at the discretion of the treating physician, with no standardized indications. Third, patient data were extracted from their electronic medical records, which resulted in a low follow-up rate, especially at 3 years. In addition, information on the symptoms was not included. Thus, we had no data regarding the proportion of patients with symptomatic heart failure. Fourth, this was a single-center study performed in Japan; thus, selection bias cannot be excluded despite the large sample size. Finally, there remain unmeasured confounders affecting long-term prognosis. Nevertheless, we conducted extensive statistical adjustment for the measured confounders.

## Conclusions

Left axis deviation was associated with a higher risk of a composite of all-cause death and MACE in patients without conduction block.

## Supplementary Information

Supplementary Information.

## Data Availability

All relevant data are within the manuscript. The raw data will be provided upon the reasonable request to the corresponding author.
