# Supplementary material for: Clinical impact of left and right axis deviations with narrow QRS complex on 3-year outcomes in a hospital-based population in Japan
Source: Sci Rep. 2021 Apr 26;11:8892. doi: 10.1038/s41598-021-88259-8 (PMC8076182; doi:10.1038/s41598-021-88259-8)
Supplement: Supplementary file 1 — Supplementary Information. [file 41598_2021_88259_MOESM1_ESM.docx]

**Supplementary Information for**

**Clinical impact of left and right axis deviations with narrow QRS complex on 3-year outcomes in a hospital-based population in Japan**

**Authors:** Yuta Seko^1^; Takao Kato^1^; Yuhei Yamaji^2^; Yoshisumi Haruna^2^; Eisaku Nakane^2^; Tetsuya Haruna^2^; Moriaki Inoko^2^

Affiliations: ^1^Department of Cardiovascular Medicine, Kyoto University Graduate School of Medicine, Kyoto, Japan ^2^Cardiovascular Center, The Tazuke Kofukai Medical Research Institute, Kitano Hospital, Osaka, Japan

Corresponding Author: Takao Kato, MD. Department of Cardiovascular Medicine, Kyoto University Graduate School of Medicine, Japan 54 Shogoin Kawahara-cho, Sakyo-ku, Kyoto. 606-8507, Japan

Tel: +81-75-751-4254; FAX: +81-75-751-3289

E-mail: [tkato75@kuhp.kyoto-u.ac.jp](mailto:tkato75@kuhp.kyoto-u.ac.jp)

**Supplementary Information content**

**Supplementary Tables Page 3-6**

**Supplementary Figures Page 7-8**

**Supplementary Figure legends Page 9**

**Supplementary Table 1: Baseline characteristics of the study subjects and transthoracic echocardiography using QRS ≥100 ms as exclusion criteria**

|  | Total  (n=2353) | Left axis deviation  (n=110) | Right axis deviation  (n=57) | Normal axis  (n=2186) | P value | Total |
| --- | --- | --- | --- | --- | --- | --- |
| Age, years | 65.5 ± 16.0 | 73.3 ± 11.5 | 60.0 ± 17.7 | 65.3 ± 16.0 | <0.001 | 2353 |
| Age >70 years* | 1083 (46.0) | 71 (64.6) | 19 (33.3) | 993 (45.4) | <0.001 | 2353 |
| Women | 1379 (58.6) | 46 (41.8) | 35 (61.4) | 1298 (59.4) | 0.001 | 2353 |
| Body mass index (BMI) kg/m^2^ | 22.8 ± 4.1 | 21.9 ± 3.6 | 21.8 ± 4.6 | 22.9 ± 4.1 | 0.008 | 2335 |
| BMI >25 kg/m^2^* | 597 (25.6) | 20 (18.5) | 9 (16.1) | 568 (26.2) | 0.053 | 2335 |
| Atrial fibrillation | 240 (10.2) | 7 (6.4) | 11 (19.3) | 222 (10.2) | 0.03 | 2353 |
| Diabetes* | 692 (29.4) | 40 (36.4) | 15 (26.3) | 637 (29.1) | 0.23 | 2353 |
| Hypertension* | 1282 (54.5) | 74 (67.3) | 21 (36.8) | 1187 (54.3) | <0.001 | 2353 |
| Dyslipidemia* | 644 (27.4) | 43 (39.1) | 10 (17.5) | 591 (27.0) | 0.005 | 2353 |
| Ischemic heart disease* | 670 (28.5) | 55 (50.0) | 18 (31.6) | 597 (27.3) | <0.001 | 2353 |
| Chronic kidney disease* | 318 (13.5) | 21 (19.1) | 6 (10.5) | 291 (13.3) | 0.18 | 2353 |
| LVDd, cm | 4.55 ± 0.52 | 4.60 ± 0.57 | 4.54 ± 0.62 | 4.54 ± 0.52 | 0.55 | 2353 |
| LVDs, cm | 3.02 ± 0.46 | 3.07 ± 0.54 | 3.05 ± 0.56 | 3.02 ± 0.45 | 0.41 | 2353 |
| IVSTd, cm | 0.79 ± 0.15 | 0.84 ± 0.18 | 0.78 ± 0.15 | 0.79 ± 0.15 | 0.004 | 2353 |
| LVPWd, cm | 0.78 ± 0.13 | 0.83 ± 0.14 | 0.75 ± 0.15 | 0.78 ± 0.13 | <0.001 | 2353 |
| RWT | 0.35 ± 0.07 | 0.36 ± 0.06 | 0.33 ± 0.07 | 0.35 ± 0.07 | 0.01 | 2353 |
| LVMI, g/m^2^ | 73.5 ± 20.7 | 82.3 ± 25.1 | 71.5 ± 24.8 | 73.1 ± 20.3 | <0.001 | 2343 |
| High LVMI* | 191 (8.2) | 17 (15.5) | 5 (8.9) | 169 (7.8) | 0.02 | 2343 |
| LAVI, ml/m^2^ | 22.8 ± 12.3 | 25.7 ± 19.4 | 24.0 ± 17.6 | 22.6 ± 11.7 | 0.047 | 2098 |
| EF, % | 62.3 ± 6.6 | 61.4 ± 7.7 | 61.3 ± 7.7 | 62.4 ± 6.5 | 0.14 | 2353 |
| EF <50 %* | 109 (4.6) | 10 (9.1) | 4 (7.0) | 95 (4.4) | 0.048 | 2353 |
| HR, bpm | 72.8 ± 15.7 | 74.3 ± 15.5 | 77.8 ± 16.6 | 72.6 ± 15.7 | 0.03 | 2353 |

*Potential risk-adjusting variables selected for cox proportional hazard model

Values are number (%), mean ± SD. P values were calculated using the chi square test or Fisher’s exact test for categorical variables, and the one-way analysis of variance (ANOVA) test for continuous variables. BMI=body mass index, EF=ejection fraction, HR=heart rate, IVSTd=diastolic interventricular septal wall thickness, LAVI=left atrial volume index, LVDd=left ventricular diastolic dimension, LVDs=left ventricular systolic dimension, LVMI=left ventricular mass index, LVPWd=diastolic left ventricular posterior wall thickness, RWT=relative wall thickness

**Supplementary Table 2. Clinical outcomes using QRS ≥100 ms as exclusion criteria**

|  | Left axis deviation  N of patients with event/N of patients at risk (Cumulative 3-year incidence [%]) | Right axis deviation  N of patients with event/N of patients at risk (Cumulative 3-year incidence [%]) | Normal axis  N of patients with event/N of patients at risk (Cumulative 3-year incidence [%]) | Variables | Unadjusted | | Adjusted | |
| --- | --- | --- | --- | --- | --- | --- | --- | --- |
|  |  |  |  |  | HR (95% CI) | P value | HR (95% CI) | P value |
| Primary outcome |  |  |  |  |  |  |  |  |
| A composite of all-cause death and MACE | 25/110 (28.0) | 8/57 (17.8) | 304/2186 (17.8) | Left axis deviation | 1.82 (1.28-2.60) | < 0.001 | 1.58 (1.10-2.27) | 0.01 |
|  |  |  |  | Right axis deviation | 0.96 (0.49-1.85) | 0.90 | 1.03 (0.53-2.00) | 0.94 |
|  |  |  |  | Normal axis | 1 (reference) |  | 1 (reference) |  |
| Secondary outcomes |  |  |  |  |  |  |  |  |
| All-cause death | 17/110 (19.2) | 6/57 (13.3) | 199/2186 (11.7) | Left axis deviation | 1.61 (1.02-2.54) | 0.04 | 1.49 (0.94-2.37) | 0.09 |
|  |  |  |  | Right axis deviation | 1.14 (0.54-2.42) | 0.73 | 1.32 (0.62-2.82) | 0.47 |
|  |  |  |  | Normal axis | 1 (reference) |  | 1 (reference) |  |
| MACE | 11/110 (13.4) | 4/57 (9.5) | 147/2186 (9.2) | Left axis deviation | 1.91 (1.16-3.14) | 0.01 | 1.43 (0.86-2.37) | 0.16 |
|  |  |  |  | Right axis deviation | 0.86 (0.32-2.32) | 0.77 | 0.93 (0.34-2.51) | 0.88 |
|  |  |  |  | Normal axis | 1 (reference) |  | 1 (reference) |  |

CI; confidence interval, HR; hazard ratio, MACE; major adverse cardiovascular events

**Supplementary Fig. 1.**

**
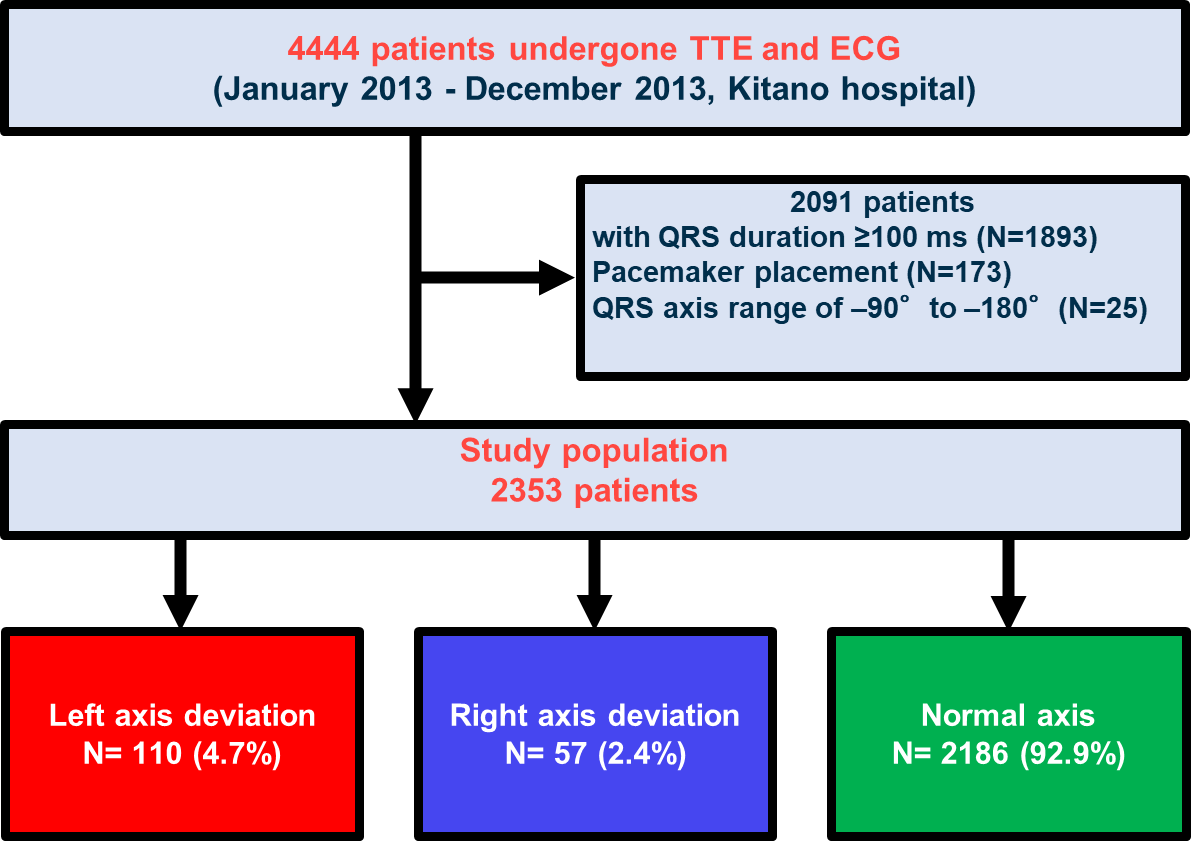
**

**Supplementary Fig. 2.**

**
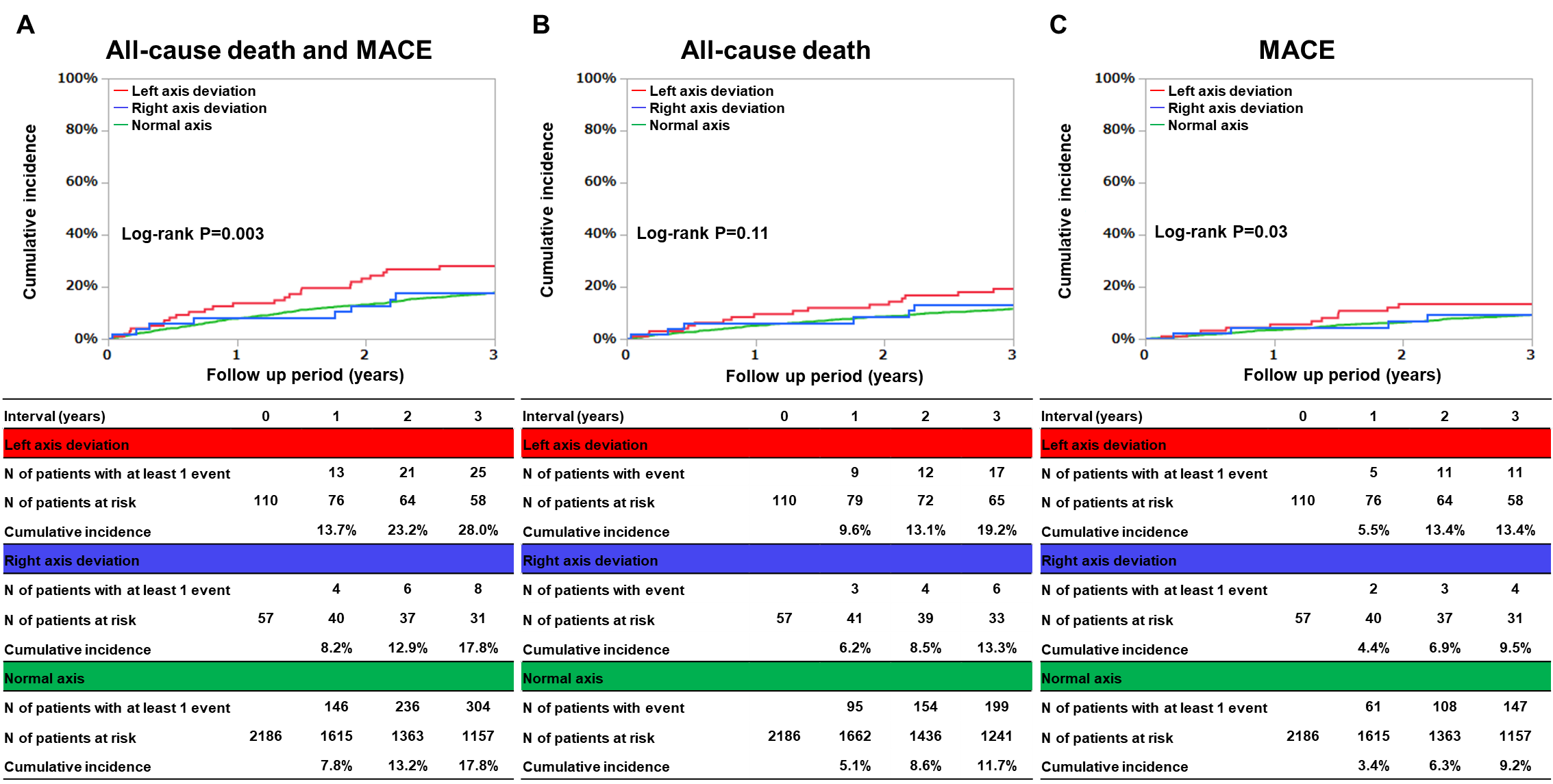
**

**Supplementary Figure legends**

**Supplementary Fig. 1.** Patient flowchart using QRS ≥100 ms as exclusion criteria. TTE=transthoracic echocardiography, ECG=electrocardiogram

**Supplementary Fig. 2**. Cumulative incidence of the primary outcome measure (a composite of all-cause death and MACE) and secondary outcomes measure (all-cause death, MACE) using QRS ≥100 ms as exclusion criteria. A; a composite of all-cause death and MACE, B; all cause death, C; MACE. MACE=major adverse cardiovascular events.
